# Supplementary material for: Single-cell sequencing of genomic DNA resolves sub-clonal heterogeneity in a melanoma cell line
Source: Commun Biol. 2020 Jun 25;3:318. doi: 10.1038/s42003-020-1044-8 (PMC7316972; doi:10.1038/s42003-020-1044-8)
Supplement: Supplementary file 1 — Supplementary Information [file 42003_2020_1044_MOESM1_ESM.pdf]

SUPPLEMENTARY INFORMATION

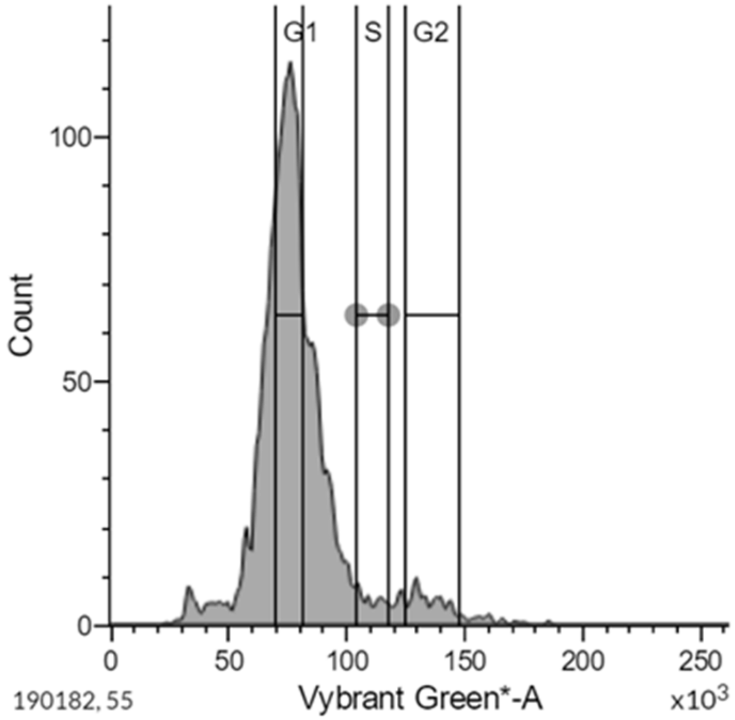

Supplementary Figure 1 | Distribution of DNA content in COLO829 nuclei by flow cytometry. Cells in the G1 phase were selected for scCNV library preparation.

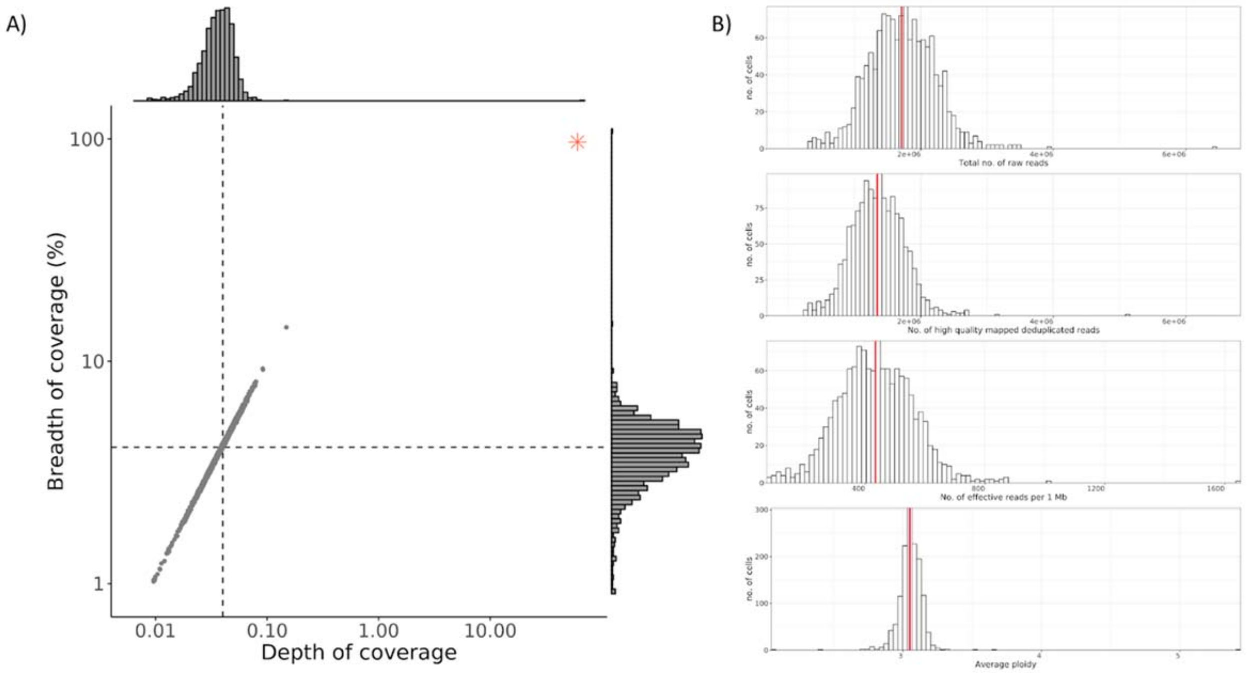

Supplementary Figure 2 | Sequencing depth . A) Depth of coverage versus breadth of coverage. A scatter plot depicted the effective depth of coverage (x-axis) versus the percent of the genome with at least 1x coverage (y-axis) for each cell. The dashed lines represent the median single cell values (0.04 depth of coverage, 4.11 breadth of coverage). The red asterix plots the values for aggregated data as a pseudo bulk experiment (60.68x depth of coverage, 95.97% breadth of coverage). **B)** Histogram of summary data quality metrics. Histogram depicting the distribution of per cell summary metrics with the median indicated by the red line: Total number of raw reads (median value 1,709,262); Number of high quality mapped deduplicated reads (median value 1,343,083); Number of effective reads per 1 Mb (median value 434) and Average ploidy (median value 3.048).

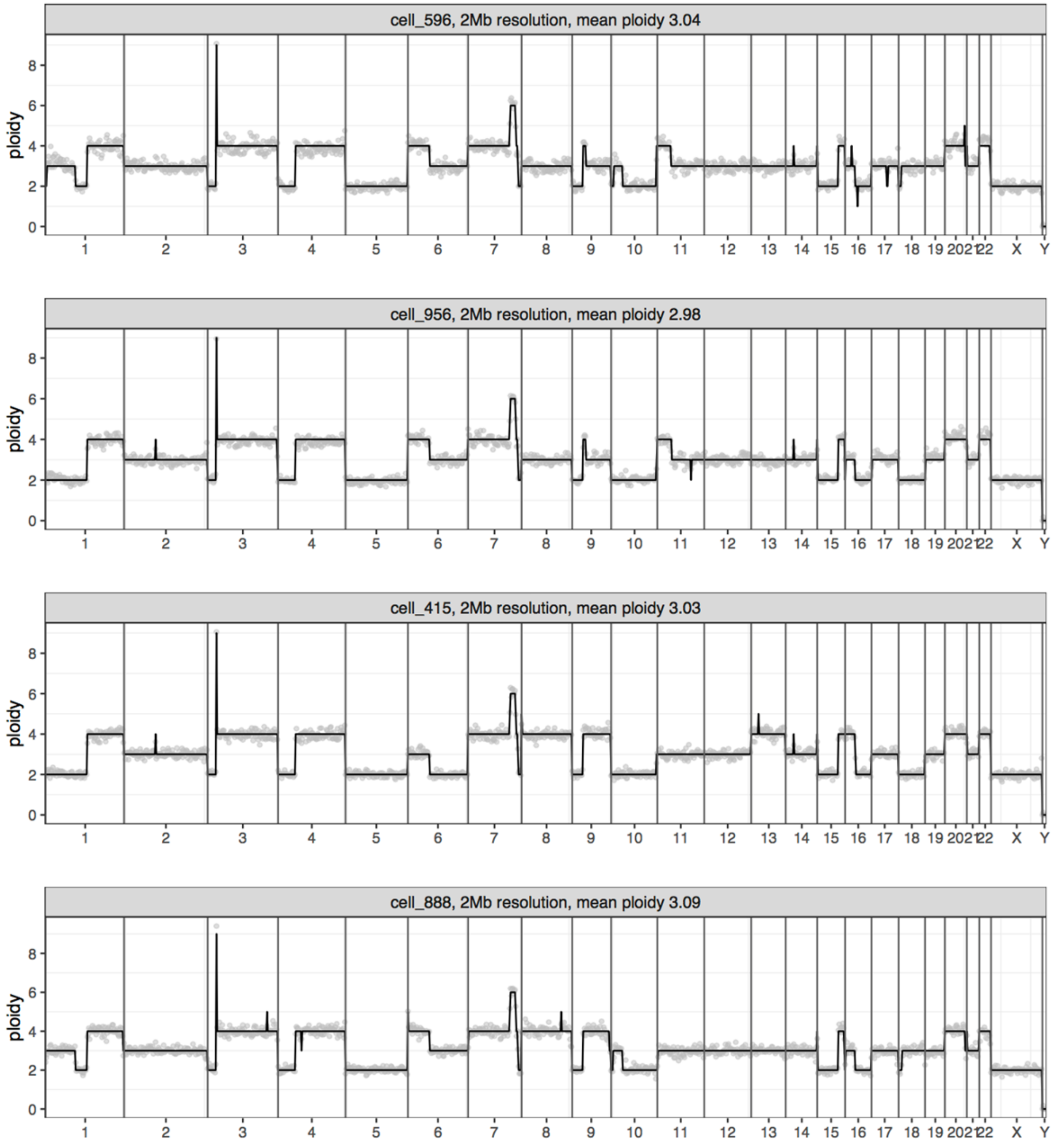

Supplementary Figure 3 | Four representative single cell ploidy plots. Copy number profiles plotted at a resolution of 2 Mb bins. Solid black line indicates ploidy call and gray dots indicate raw read counts for each bin.

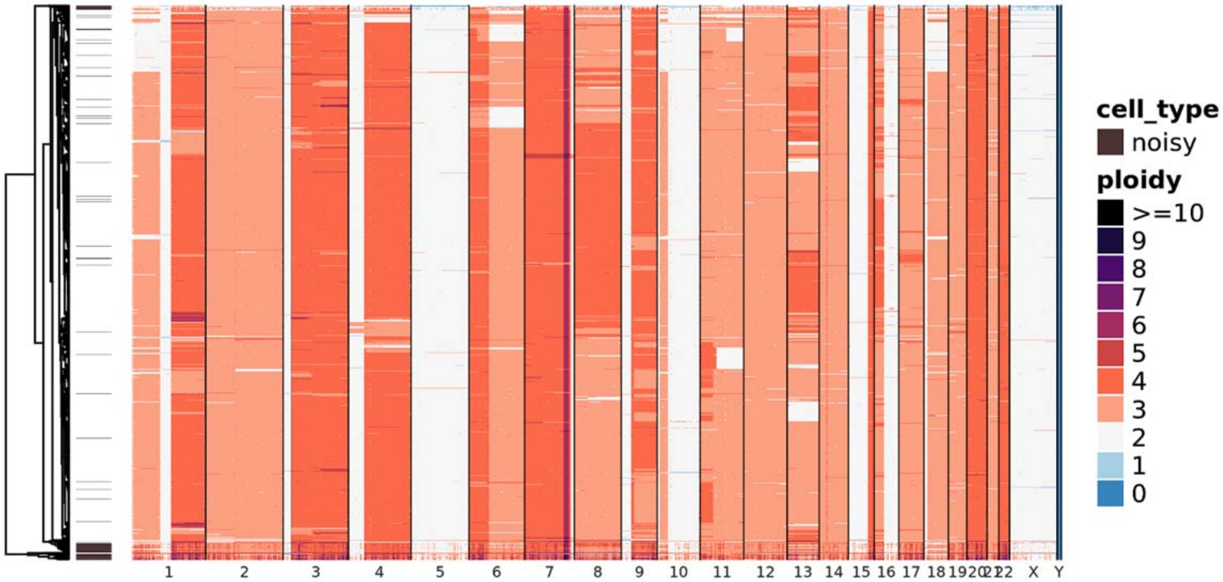

Supplementary Figure 4 | Heatmap of raw data. Heatmap showing hierarchical clustering of 1,475 single cell CNV profiles at 2 Mb resolution. Each row depicts the whole genome of a single cell and colors represent the called ploidy as specified by the legend on the right. Color-bar on the right marks single cells labeled by the pipeline as noisy in brown.

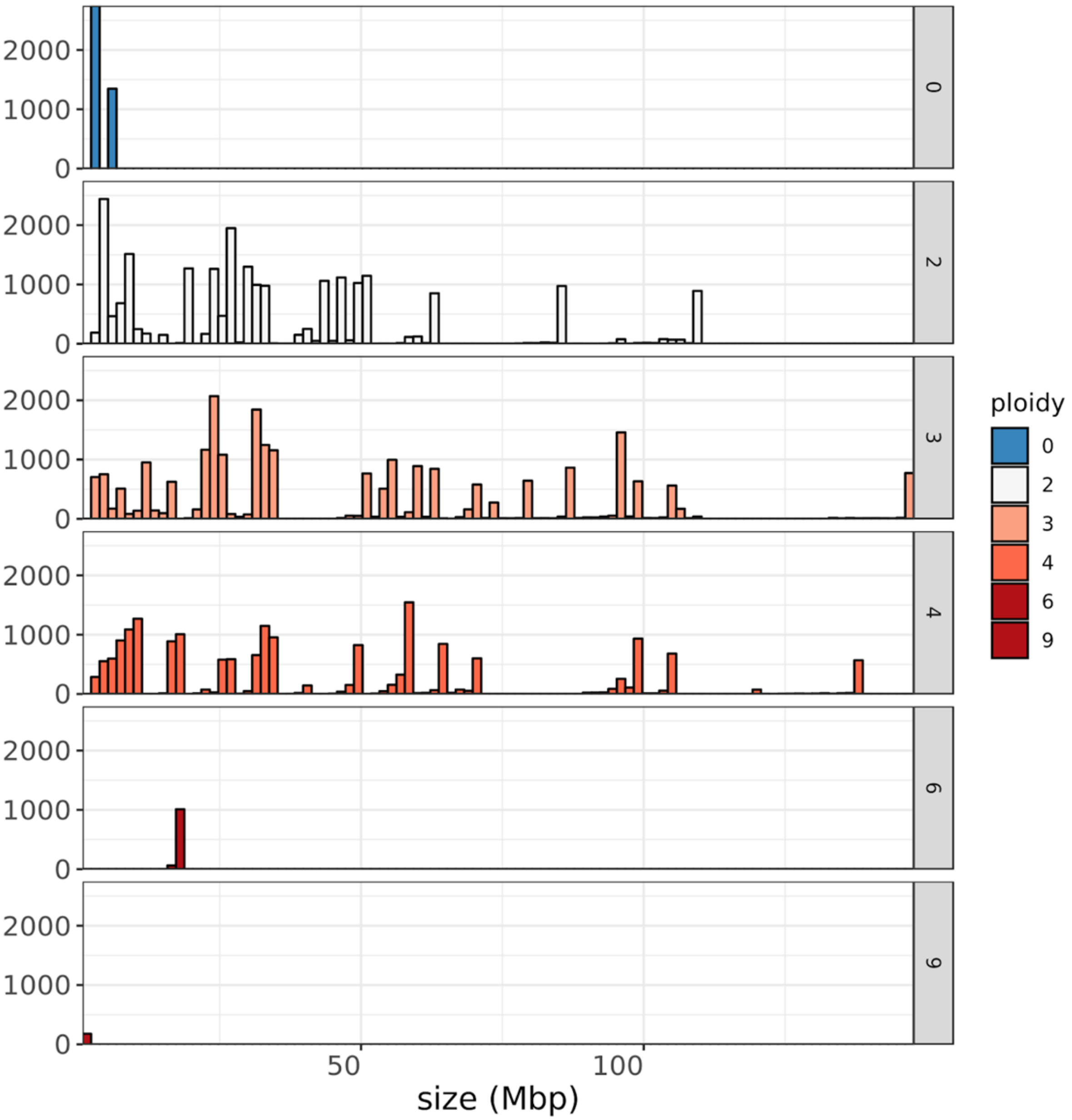

Supplementary Figure 5 | Size distribution of CNV events by ploidy. Histogram of the 114 CNV events that passed the following filtering criteria: event quality > 15; event size > 2 Mb and event frequency > 0.05.

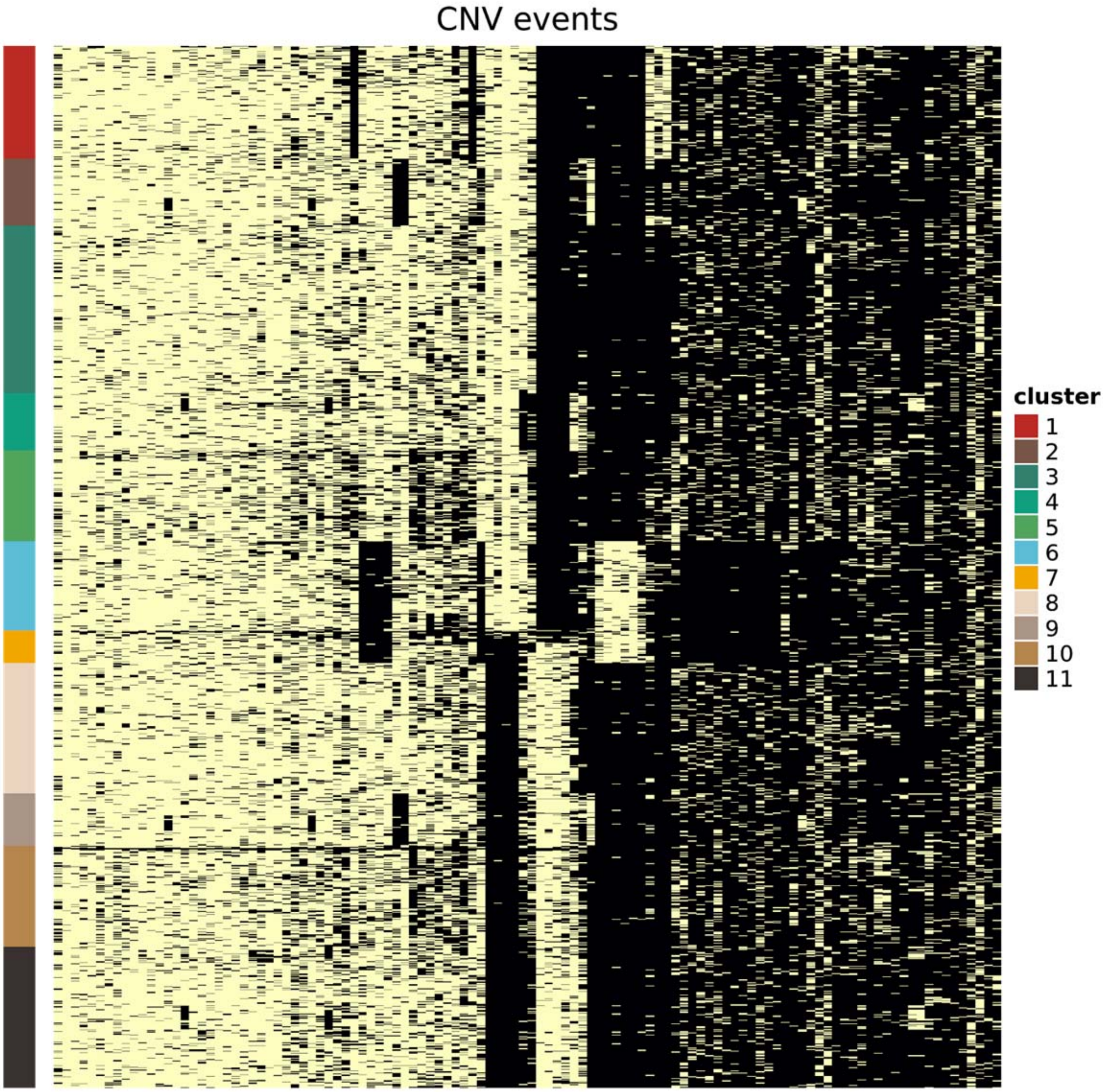

Supplementary Figure 6 | CNV events within each cluster. Heatmap with one row for each cell and one column for each of the 122 polymorphic CNV events that passed filtering, ordered by their frequency in the population. The cells are ordered by membership in DAPC clusters. The presence or absence of a mutation is depicted in yellow and black, respectively.

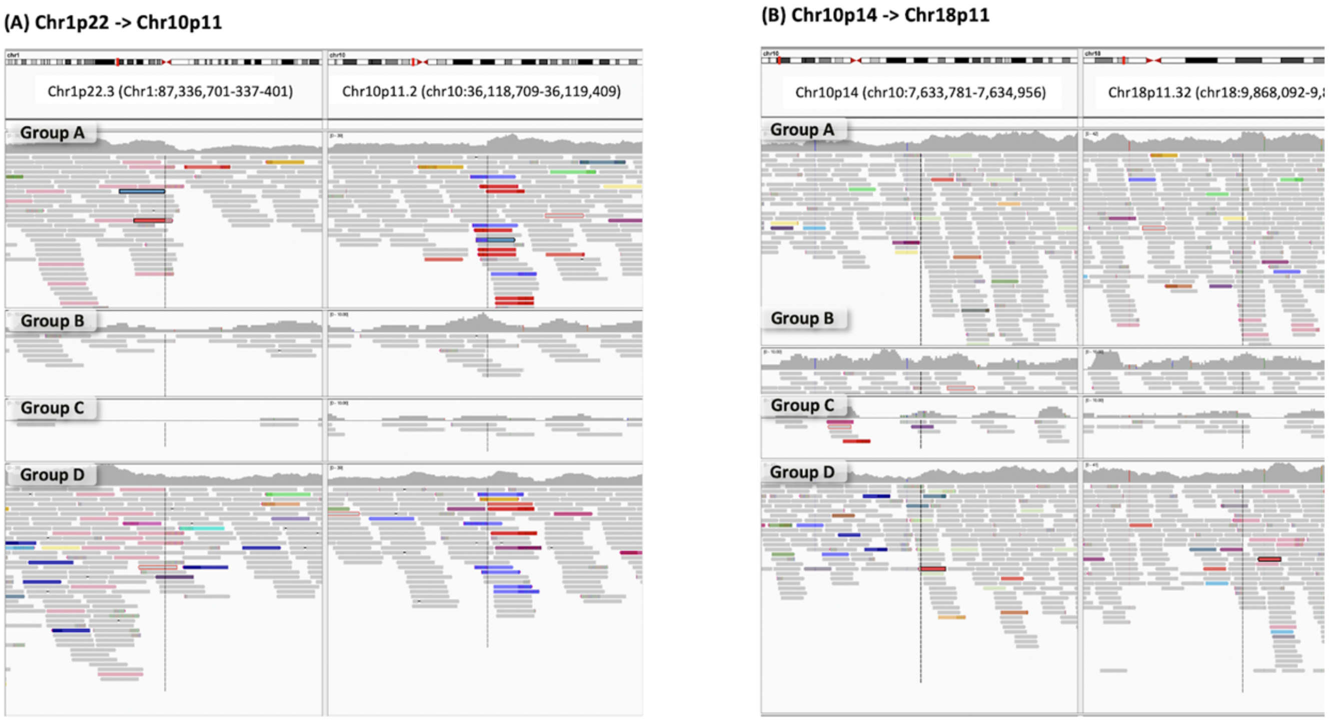

Supplementary Figure 7 | IGV Traces of anomalous reads spanning translocations. Read coloring is formally defined within the IGV manual and are set to indicate instances where the insert distances between paired reads is beyond 1kb. Reads mapping within the same chromosome are red, reads mapping to chromosome 18 are in pink, reads mapping to chromosome 10 are blue, reads mapping to chromosome 1 are light green. **(Left)** Split view of Chr1p22 <-> Chr10p11. Reads in pink are those whose partner maps to chr10p11, whereas reads whose partner maps to chr1p22 are shown in blue. Reads in red read map 2.7Mb upstream. **(Right)** Split view of Chr10p14 <-> chr18p11. Pink reads indicate where the partner maps to Chr18p11.32. Light green reads indicate where the read partner maps to chromosome 10p11.

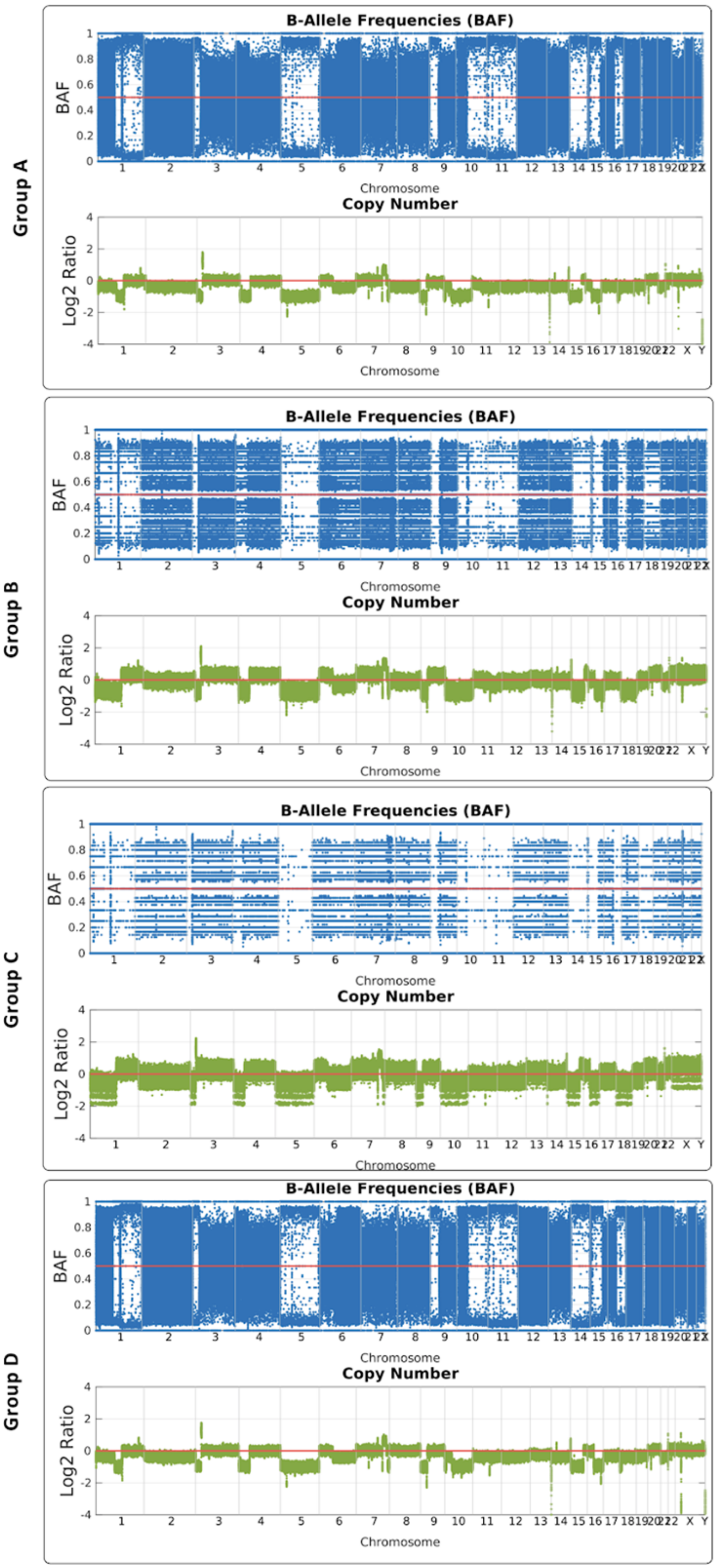

Supplementary Figure 8 | Log2(Fold Change) and LOH for each group. The lower graphs show the Log2(Fold Change) using the program tCoNuT, which was used previously for bulk sequencing and produces allele fraction plots. It is important to note that tCoNuT, like other bulk copy number tools cannot determine copy number natively without assumptions for deconvoluting potential mixtures. Single cell copy numbers as used elsewhere do not require this assumption and absolute ploidy can be better inferred. The upper graphs provide the allele fractions of known heterozygous SNPs determined previously from sequencing on the lymphoblastic germline pair. For example in *Group A* at chr5 when copy number is 1, we see allele fractions partitioning into 0 and 1 allele fractions indicating homozygosity. The relative noise is dependent on number of reads over a SNP, and thus greater spread is shown. In the figure above we observe for chromosomes 5, 10, 11 and 14. We also see some chromosomes with partial LOH. Within the main figure 4, chromosome 1, 10, and 18 are shown, whereas this figure shows all chromosomes.

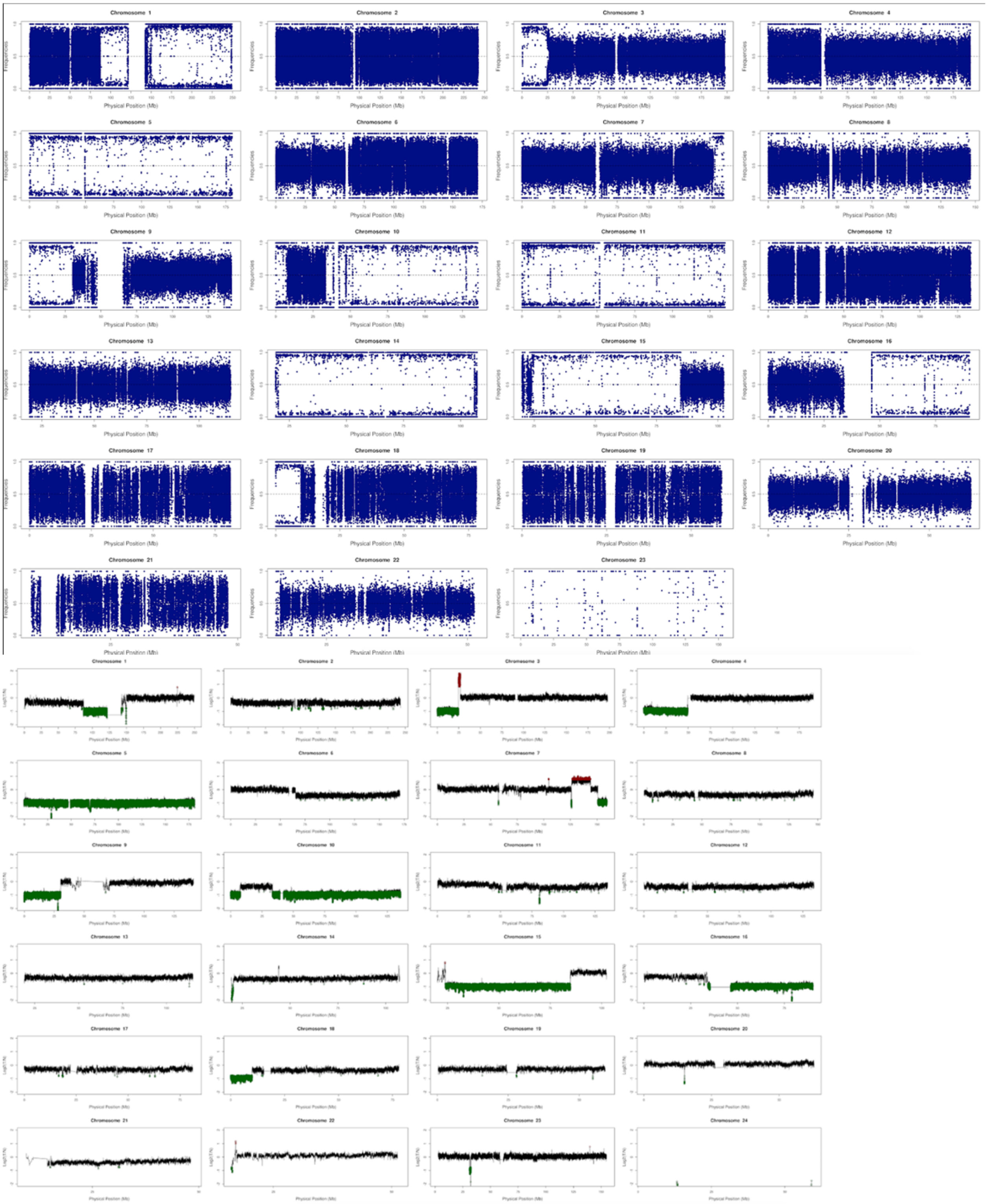

Supplementary Figure 9 | Exemplary Loss of Heterozygosity (LOH) broken out by chromosome for Group A (upper) and copy number estimate shown by chromosome.

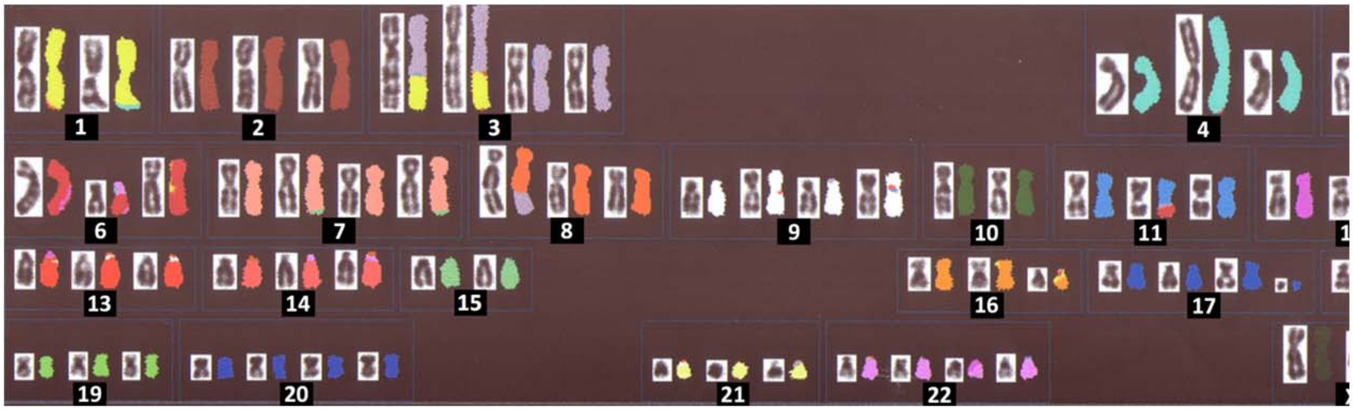

Supplementary Figure 10 | Spectral Karyotype (SKY) of COLO829 cell-line. SKY multicolour-FISH Karyotype from 10 metaphases, performed without knowledge of the single cell sequencing and from a separate sample of the cell line, as described[10]. The karyotype can be regarded as pseudo-tetraploid with losses. It shows clear signs of endoreduplication, several abnormal chromosomes being present in two copies. There is evidence of Dutrillaux's monosomic pattern of karyotype evolution with endoreduplication - e.g. 2 copies each of chromosomes 1, 3 and an unbalanced 1;3 translocation; 2 each of 7, 15 and a probable 7;15 translocation. Consensus karyotype: 69 chromosomes (mode 68) 2 x 1, 2 x 3, 3 x 2, der(?)t(1;3)(q?;p22-24?) x 2, 4 x 2, iso(4), 5 x 2, 6 x 2, del(6) x 2, 7 x 2, der(7)t(7;15?*) x 2, 8 x 3, 9 x 2, del(9) x 2, 10 x 2, 11 x 3**, 12 x 3, 13 x 3, 14 x 3, 15 x 2, 16 x 2, del(16), 17 x 3, 18 x 2, 19 x 3, 20 x 4, 21 x 3, 22 x 4, X x 2.

* the fragment is identified by SKY as chromosome 15 but the fragment is very small so this identification is unreliable.
** In 6/10 metaphases there are 3 elevens. In four of these there is an additional unbalanced 1;18 translocation, der(?)t(1p?;18q?), while in the other 4 metaphases one eleven is replaced by a der(?)t(11;18). A possible explanation is that the consensus should include the 1;18 translocation and three elevens, but in some metaphases one eleven and the 1;18 have combined to yield the single der(?)t(11;18).

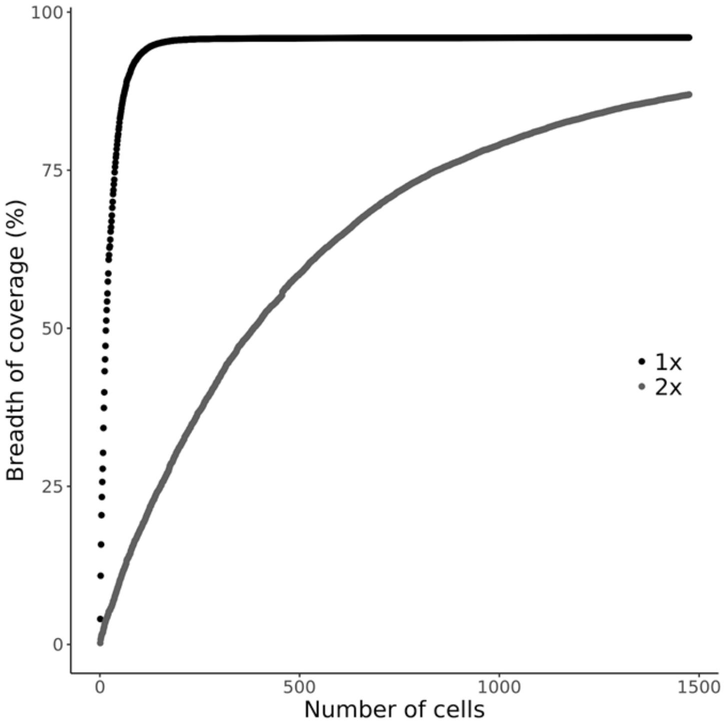

Supplementary Figure 11 | Cumulative breadth of coverage. A cumulative plot of genome coverage at two different depths 1x and 2x (black and gray, respectively). Number of cells needed to achieve 75% coverage across the genome is 37 at 1x depth and 845 at 2x depth.

Supplementary Table 1 | Aggregate summary metrics. Summary metrics describing sequencing data quality by group.

Groups	Number of cells	Number of reads	Number of effective reads	Average ploidy	Median reads per Mb
**A**	653	1,106,320,742	866,039,383	3.03	428
**B**	117	195,118,990	152,584,425	2.95	416
**C**	43	78,145,878	60,875,291	3.01	450
**D**	560	975,448,264	763,375,990	3.11	435

Supplementary Code 1 | R script to perform Clustering of single cell CNV data, generating a CNV mutation matrix with polymorphic events.

```
library(GenomicRanges)
library(dplyr)
# path to outs folder
PATH_2_OUTS <- "YOUR_PATH"
# read the per cell summary
per_cell_summary <-
read.table(file.path(PATH_2_OUTS,"per_cell_summary_metrics.csv"),
sep=",",header = TRUE)
# define inputs and cutoffs
cells <- per_cell_summary$cell_id[per_cell_summary$is_noisy==0]
cells <- paste0("cell_",cells)
NCELLS <- length(cells)
EVENT_QUAL = 15
EVENT_SIZE = 2e6
EVENT_FREQ = .05
CUTOFF_N <- round(NCELLS*EVENT_FREQ)
#' read bed file and subset to keep events only from
#'terminal nodes that are not flagged as noisy
get_bed <- function(path_2_outs){
  df <-
read.table(file.path(path_2_outs,"node_cnv_calls.bed"),header=FALSE,sep="\t")
  names(df) <- c("chrom", "start", "end", "node", "ploidy", "qual")
  nnodes <- max(df$node)
  ncells <- nnodes/2
  df$node <-
ifelse(df$node>ncells,paste0("inode_",df$node),paste0("cell_",df$node))
  return(df)
}
bed <- get_bed(PATH_2_OUTS)
bed <- subset(bed,node %in% cells)
bed.gr <- makeGRangesFromDataFrame(bed, keep.extra.columns=TRUE,
                                   ignore.strand=TRUE,
                                   seqinfo=NULL,
                                   seqnames.field="chrom",
                                   start.field="start",
                                   end.field="end",
                                   starts.in.df.are.0based=TRUE)
# apply a quality and size cut off and
# order the events going from largest to smallest
bed.gr <- bed.gr[bed.gr$qual>EVENT_QUAL]
bed.gr <- bed.gr[width(bed.gr) > EVENT_SIZE]
bed.gr <- bed.gr[order(-width(bed.gr),start(bed.gr))]
#split bed file by ploidy and chromosome
bed.gr <- split(bed.gr,bed.gr$ploidy)
split.bed.gr <- lapply(bed.gr, function(x) split(x,seqnames(x)))
# find pairwise 90% reciprocal overalps with the same ploidy
events.byploidy <- list()
for (i in seq_along(split.bed.gr)){
  gr <- split.bed.gr[[i]]
  hits <- lapply(gr, function(x)
findOverlaps(x,drop.self=FALSE,drop.redundant=TRUE,type="any",select="all"))
  xhits <- lapply(seq_along(gr), function(x) gr[[x]][queryHits(hits[[x]])])
  yhits <- lapply(seq_along(gr), function(x) gr[[x]][subjectHits(hits[[x]])])
  pmax <- lapply(seq_along(gr), function(x)
pmax(width(yhits[[x]]),width(xhits[[x]])))
  overlaps <- lapply(seq_along(gr), function(x)
pintersect(xhits[[x]],yhits[[x]]))
```

```
  frac <- lapply(seq_along(gr), function(x) width(overlaps[[x]])/pmax[[x]])
  merge <- lapply(frac, function(x) x>=0.9)

  final <- list()
  for(j in seq_along(gr)){
    tokeep <- yhits[[j]][merge[[j]]]
    tokeep$qhit <- queryHits(hits[[j]])[merge[[j]]]
    tokeep$subhit <- subjectHits(hits[[j]])[merge[[j]]]
    #the same subject hit is being grouped multiple times and supporting
different queries
    tokeep <- tokeep[!duplicated(tokeep$subhit)]
    qcount <- table(tokeep$qhit)
    tokeep <- tokeep[tokeep$qhit %in% names(qcount[qcount>CUTOFF_N])]
    final[[j]] <- tokeep
  }
  final <- do.call("c",final)
  events.byploidy[[i]] <- final
}

# generate unique labels for shared events
label.qhit <- sapply(events.byploidy, function(x) paste0(x$qhit))
label.chr <- sapply(events.byploidy, function(x) as.vector(seqnames(x)))
label <- sapply(seq_along(label.qhit), function(i)
paste0(label.chr[[i]],label.qhit[[i]]))
label <- sapply(seq_along(label),function(i) paste0("ploidy",i-
1,"_chr",label[[i]]))
label <- unlist(label)
tokeep <- sapply(label,function(x) grepl("ploidy\\d+_chr[XY]*\\d+",x))
label <- label[tokeep]
label <- factor(label,labels = 1:length(unique(label)))

# make a single GenomicRanges object
events.05.gr <- do.call("c",events.byploidy)
events.05.gr$event <- label
events.05.gr$qhit <- NULL
events.05.gr$subhit <- NULL
rm(events.byploidy)
```

Supplementary Code 2 | R script to aggregate matrix of CNV events with customized bin-sizes.

```
#' An aggregated matrix of CNV events with customized bin-sizes can be
generated
#' using the cnv_data.h5 file in the outs folder of a Cellranger DNA
pipestance.
#' A boolean vector specifying the mappability of all 20-kb bin across each
chromosome
#' can be extracted from the "/genome_tracks/is_mappable" field of the h5
file.
#'
#' This vector is then used to determine the number of 20-kb bins in each
chromosome
#' and their genomic coordinates. The CNV values are contained within the
"/cnvs" field
#' of the same h5 file. This loads up a list of matrices split by chromosome
where the
#' rows are the 20-kb bins and the columns are all nodes (terminal + internal
nodes).
#' The values of the CNV calls contain custom keys specifying imputation etc
the details
#' of whcih are available here: https://support.10xgenomics.com/single-cell-
dna/software/pipelines/latest/output/hdf5.
#'
#' This initial matrix tends to be large because the number of rows is ~
genome size/20-kb.
#' For plotting, bins can be aggregated to a more manageable resolution. For
example,
#' averaging the CNV counts from adjacent 100 bins will result in a CNV event
matrix of 2 Mbp resolution.

require(rhdf5)
require(GenomicRanges)
require(ComplexHeatmap)

#--------GENERATE AN AGGREGATED CNV MATRIX-----------------------------------
--------------

# path to outs folder
PATH_2_OUTS <- "path_to_your_pipestance_outs_folder"

# define the input variables
RES <- 100 # No. of 20kb bins to aggregate

make_granges <- function(contig, nbins){
  start <- as.character((0:(nbins-1))*20000)
  end <- as.character((1:nbins)*20000)
  ranges <- paste(contig,":",start,"-",end,":*")
  return(ranges)
}

#' Read the is_mappability track i.e. for each chromosome and generate
#' a named genomic range for each bin
data <-
rhdf5::h5read(file.path(PATH_2_OUTS,"cnv_data.h5"),"/genome_tracks/is_mappabl
e")
bins_per_chrom <- sapply(data, length)
chroms <- names(data)
ranges <- mapply(make_granges, chroms, bins_per_chrom, SIMPLIFY = TRUE)
ranges <- unlist(ranges)
```

```
is_mappable <- as.logical(unlist(data))

#' Read the cnv track which has for each chromosome a matrix with rows
#' as the bins and columns as all nodes i.e. terminal observed cells as well
#' as internal nodes of the heirarchical clustering tree. The values are CNV
#' calls with custom keys defining imputed values, etc details of which are
described here
#' (https://support.10xgenomics.com/single-cell-
dna/software/pipelines/latest/output/hdf5).
data <- rhdf5::h5read(file.path(PATH_2_OUTS,"cnv_data.h5"),"/cnvs")
data <- do.call(rbind,data)
data[data==-127] <- 0
data[data==-128] <- NA
data <- abs(data)

nnodes <- dim(data)[2]
ncells <- (nnodes+1)/2
rownames(data) <- ranges

#' Subset the CNV matrix to contain only rows that correspond to mappable
bins
#' and columns that are terminal nodes
data <- data[is_mappable,1:ncells]
colnames(data) <- paste0("cell_",0:(ncells-1))

#' Convert into a genomic ranges object
gr <- GenomicRanges::GRanges(rownames(data))
GenomicRanges::mcols(gr) <- data
rm(data, bins_per_chrom,chroms,is_mappable,nnodes,ranges, make_granges)

#' Split the genomic ranges object by chromosomes and define the
#' genomic coordinates of the aggregate bins at the desired resolution
#' for e.g. 2 Mb bins represents ploidy averaged across 100 20kb bins.
#' Do this by generating an aggregation vector i.e. across all 20 kb bins
#' a numeric classifiation of which bins are going to be collapsed.
gr <- GenomeInfoDb::sortSeqlevels(gr)
gr <- sort(gr)
gr_list <- split(gr,GenomicRanges::seqnames(gr))
nbins_20kb <- sapply(gr_list,function(x) length(x))
nbins_aggr <- sapply(nbins_20kb, function(x) ceiling(x/RES)) # no of
aggregated bins per chromosome
chrom_bdry <- c(0,cumsum(nbins_aggr))
chrom_bdry <- chrom_bdry + 1
names(chrom_bdry) <- c(names(chrom_bdry)[-1],"") # boundary bins
aggr <- sapply(1:length(nbins_aggr), function(x)
rep(chrom_bdry[x]:chrom_bdry[x+1],each=RES)[1:nbins_20kb[x]])
aggr <- unlist(aggr)
rm(gr_list, nbins_20kb)

#' Calculate the mean ploidy values grouped by the aggregate vector
#' for each cell and round them out.
m = GenomicRanges::mcols(gr)
metadata <- aggregate(.~aggr,m,mean)

metadata$aggr <- NULL
metadata[] <- lapply(metadata, round)

#' Locate the genomic co-ordinates of the aggregated bins
unik <- !duplicated(aggr)  ## logical vector of unique values
bin_start <- seq_along(aggr)[unik]  ## indices
bin_end <- bin_start - 1
```

```
bin_end <- bin_end[-1]
bin_end <- c(bin_end,length(aggr))

#' generate a aggregated genomic ranges object
df = data.frame(chrom=GenomicRanges::seqnames(gr)[bin_start],

start=GenomicRanges::start(GenomicRanges::ranges(gr))[bin_start],
                stop=GenomicRanges::end(GenomicRanges::ranges(gr))[bin_end])
df = cbind(df,metadata)

#generate a granges object
gr_aggr <- GenomicRanges::makeGRangesFromDataFrame(df,
keep.extra.columns=TRUE,ignore.strand=TRUE)
rm(aggr, m, metadata, unik, bin_start, bin_end, df)

#-----------PLOT THE CNV MATRIX AS A HEATMAP---------------------------

#' plot a heatmap with chromosome boundaries
#' the order of the rows can be customized here
#' its a simple distance based clustering

mat <- as.matrix(GenomicRanges::mcols(gr_aggr))
mat <- t(mat)
hr <- hclust(dist(mat), method = "average")
hr = as.dendrogram(hr)

# additional chromosome annotations
nbins <- chrom_bdry[length(chrom_bdry)]
abline_x = chrom_bdry[-1]/nbins
x_label <- names(nbins_aggr)
mid.point <- (nbins_aggr/2)/nbins
mid.point <- abline_x - mid.point
abline_ids <- rep(1:length(abline_x),each=2)
abline_y <- rep(c(0,1), times=length(abline_x))
rownames(abline_x) <- NULL
abline_x <- rep(abline_x,each=2)
rm(nbins)

#annotation to label chromosomes
ha_column = ComplexHeatmap::HeatmapAnnotation(cn = function(index) {
  grid.text(x_label,x= mid.point,y=1,just = c("center",
"top"),gp=gpar(col="#202020",fontsize=6))
})

#the main heatmap
mat[mat>=6] = ">=6"
colors =
structure(rev(c("#3783bb","#a7d0e4","#f7f6f6","#fca082","#fb694a","#e32f27","
#b11218")),
                   names = rev(c("0", "1", "2", "3","4","5",">=6")))

hm = ComplexHeatmap::Heatmap(matrix = mat,
                 name = "ploidy",
                 col = colors,
                 cluster_rows = hr,
                 cluster_columns = FALSE,
                 show_row_names = FALSE,
                 bottom_annotation = ha_column,
                 column_title = "CNV heatmap @ 2Mb resolution")

ComplexHeatmap::draw(hm, row_dend_side = "left")
```

```
ComplexHeatmap::decorate_heatmap_body("ploidy", {
  grid.polyline(x= abline_x,
                y = abline_y,
                id = abline_ids,
                gp = gpar(lty = 1, lwd = 1.5))
})
```

Supplementary Code 3 | Python script to split BAM files by barcode assignment, generating a BAM file for each sub-clone.

```
import pysam
import pandas as pd
import sys
import os
import argparse

def parse_args():
  parser = argparse.ArgumentParser()
parser.add_argument('--bam', help='Input BAM file')
parser.add_argument('--out-root', help='Root directory to write output
BAMs to')
parser.add_argument('--csv', help='CSV file with two columns: barcode
and cluster.')
return parser.parse_args()

if __name__ == '__main__':
  args = parse_args()
df = pd.read_csv(args.csv)
assert 'barcode' in df.columns and 'cluster' in df.columns, 'Missing
column identifiers'
assert os.path.exists(args.bam), 'Cannot find input BAM'
fh = pysam.Samfile(args.bam)
cluster_map = dict(zip(df.barcode, df.cluster))
os.makedirs(args.out_root)
out_handles = {}
for c in set(df.cluster):
  o = pysam.Samfile(os.path.join(args.out_root, 'cluster' + str(c) +
'.bam'), 'wb', template=fh)
out_handles[c] = o
for rec in fh:
  if rec.has_tag('CB') and rec.get_tag('CB') in cluster_map:
  cluster = cluster_map[rec.get_tag('CB')]
out_handles[cluster].write(rec)
for h in out_handles.values():
  h.close()
```
