# Supplementary material for: Single-cell sequencing of genomic DNA resolves sub-clonal heterogeneity in a melanoma cell line
Source: Commun Biol. 2020 Jun 25;3:318. doi: 10.1038/s42003-020-1044-8 (PMC7316972; doi:10.1038/s42003-020-1044-8)
Supplement: Supplementary file 2 — Description of Additional Supplementary Items [file 42003_2020_1044_MOESM2_ESM.pdf]

Description of additional Supplementary Files

Supplementary Data 1 (Excel file)

Per cell summary metrics. Per cell metrics describing sequencing and CNV estimation data quality.

Supplementary Data 2 (Excel file)

DAPC classification of single cells. Assignment of each barcode to DAPC defined cluster and group.

Supplementary Data 3 ((Excel file).

CNV events by cluster. Frequency of each of the CNV events that passed filters across the 11 clusters and 4 groups. The start and end headers are the median values of the edges shared across all single cells for that event.
