# Supplementary material for: Single-cell sequencing of genomic DNA resolves sub-clonal heterogeneity in a melanoma cell line
Source: Commun Biol. 2020 Jun 25;3:318. doi: 10.1038/s42003-020-1044-8 (PMC7316972; doi:10.1038/s42003-020-1044-8)
Supplement: Supplementary file 6 — Reporting Summary [file 42003_2020_1044_MOESM6_ESM.pdf]

nature research

Corresponding author(s): Enrique I. Velazquez-Villarreal, MD, PhD
David W. Craig, PhD

Last updated by author(s): Feb 29, 2020

Reporting Summary

Nature Research wishes to improve the reproducibility of the work that we publish. This form provides structure for consistency and transparency in reporting. For further information on Nature Research policies, see Authors & Referees and the Editorial Policy Checklist.

Statistics

For all statistical analyses, confirm that the following items are present in the figure legend, table legend, main text, or Methods section.

n/a	Confirmed	
☐	☒	The exact sample size (*n*) for each experimental group/condition, given as a discrete number and unit of measurement
☐	☒	A statement on whether measurements were taken from distinct samples or whether the same sample was measured repeatedly
☐	☒	The statistical test(s) used AND whether they are one- or two-sided<br>*Only common tests should be described solely by name; describe more complex techniques in the Methods section.*
☐	☒	A description of all covariates tested
☐	☒	A description of any assumptions or corrections, such as tests of normality and adjustment for multiple comparisons
☐	☒	A full description of the statistical parameters including central tendency (e.g. means) or other basic estimates (e.g. regression coefficient) AND variation (e.g. standard deviation) or associated estimates of uncertainty (e.g. confidence intervals)
☐	☒	For null hypothesis testing, the test statistic (e.g. *F*, *t*, *r*) with confidence intervals, effect sizes, degrees of freedom and *P* value noted<br>*Give P values as exact values whenever suitable.*
☐	☒	For Bayesian analysis, information on the choice of priors and Markov chain Monte Carlo settings
☐	☒	For hierarchical and complex designs, identification of the appropriate level for tests and full reporting of outcomes
☐	☒	Estimates of effect sizes (e.g. Cohen's *d*, Pearson's *r*), indicating how they were calculated

Our web collection on statistics for biologists contains articles on many of the points above.

Software and code

Policy information about availability of computer code

Data collection	Paired-end reads were processed using version 1.0 of the cellranger-dna pipeline (10x Genomics), please see references 8 and 9. The pipeline consists of barcode processing, alignment to the (hg19) genome and the identification of cell-associated barcodes. Copy number calling is performed on each barcode separately after masking out regions of the genome with low mappability and normalizing for GC content. 1,475 barcodes were defined as cells, roughly all barcodes with greater than 1/10th the number of reads as the maximum per-barcode read count. Cells flagged as noisy by the pipeline (102 cells, 6.9%) were removed from downstream analysis, leaving behind 1,373 cells
Data analysis	Single cell CNV calls were extracted from a BED file generated by the cellranger-dna pipeline for 1,373 cell barcodes. Custom R script used to perform Clustering of single cell CNV data analysis is included as Supplementary File 1. To identify clusters we implemented the fast maximum-likelihood (ML) genetic clustering and Bayesian Information Criterion (BIC), subsequently, using the Bioconductor adegenet package (version 2.1.1). For Bulk Copy Number and Loss of Heterozygosity analysis, a python script was used to split the BAM file by barcode assignment, generating a BAM file for each sub-clone (please see Supplementary File 2). With cellranger-dna version 1.1 this functionality is a new sub-pipeline. Fold change and Loss of Heterozygosity (LOH) analysis was performed using previously developed tools, tCoNuT(1.0) (https://github.com/tgen/tCoNuT) and DNACopy (version 1.48.0) for both the single cell grouped BAMs, and for previous bulk sequencing of COLO829 growths. heterozygous germline variants were identified using GATK's HaplotypeCaller (version 3.5.0) from previous sequencing data. The VCFs were annotated with dbSNP 147 using snpEff (version 3.5h) and input to the tCoNuT parseMergeVCF script (1.0). For Variant detection and breakpoint analysis, we utilized a previously validated script for detection of anomalous read pairs (tgen_somaticSV) to identify clusters of read-pair mappings consistent with translocations, inversions, and other structural variants<br>(https://github.com/davcraig75/tgen_somaticSV). We attempted to identify point mutations specific to clones using Strelka2.

For manuscripts utilizing custom algorithms or software that are central to the research but not yet described in published literature, software must be made available to editors/reviewers. We strongly encourage code deposition in a community repository (e.g. GitHub). See the Nature Research guidelines for submitting code & software for further information.

Data

Policy information about availability of data

All manuscripts must include a data availability statement. This statement should provide the following information, where applicable:

- Accession codes, unique identifiers, or web links for publicly available datasets
- A list of figures that have associated raw data
- A description of any restrictions on data availability

DATA AVAILABILITY. Data have been deposited in the following 10X Genomics hosted link: https://support.10xgenomics.com/single-cell-dna/datasets/1.0.0/colo829_G1_1k.

Field-specific reporting

Please select the one below that is the best fit for your research. If you are not sure, read the appropriate sections before making your selection.

☒ Life sciences ☐ Behavioural & social sciences ☐ Ecological, evolutionary & environmental sciences

For a reference copy of the document with all sections, see nature.com/documents/nr-reporting-summary-flat.pdf

Life sciences study design

All studies must disclose on these points even when the disclosure is negative.

Sample size	We performed shallow single-cell sequencing of genomic DNA across 1,475 cells from a well-studied cell-line, COLO829, to resolve overall tumor complexity and clonality. As described previously, the pipeline consists of barcode processing, alignment to the (hg19) genome and the identification of cell-associated barcodes. Copy number calling is performed on each barcode separately after masking out regions of the genome with low mappability and normalizing for GC content. 1,475 barcodes were defined as cells, roughly all barcodes with greater than 1/10th the number of reads as the maximum per-barcode read count. Cells flagged as noisy by the pipeline (102 cells, 6.9%) were removed from downstream analysis, leaving behind 1,373 cells.
Data exclusions	1,475 barcodes were defined as cells, roughly all barcodes with greater than 1/10th the number of reads as the maximum per-barcode read count. Cells flagged as noisy by the pipeline (102 cells, 6.9%) were removed from downstream analysis, leaving behind 1,373 cells.
Replication	All findings can be verified and reproduced, this is a well-studied cell-line and methods and coding are sharing through supplemental files and through pointed references. Data is publicly available.
Randomization	COLO829 cell line was obtained from American Type Culture Collection (ATCC), Manassas, VA. Cells were cultured in their recommended media conditions. Single-cell suspension was processed using Chromium Single-Cell CNV Solution (10x Genomics) as described in the user guide to generate a barcoded single cell DNA library (Fig. 1A). Methods and code are sharing through supplemental files and through pointed references
Blinding	We performed shallow single-cell in this melanoma tumor-line that has been previously characterized by multiple technologies and provides a benchmark for evaluating somatic alterations, thus blinding was not relevant in our study.

Reporting for specific materials, systems and methods

We require information from authors about some types of materials, experimental systems and methods used in many studies. Here, indicate whether each material, system or method listed is relevant to your study. If you are not sure if a list item applies to your research, read the appropriate section before selecting a response.

Materials & experimental systems

n/a	Involved in the study	
☒	☐	Antibodies
☐	☒	Eukaryotic cell lines
☒	☐	Palaeontology
☒	☐	Animals and other organisms
☒	☐	Human research participants
☒	☐	Clinical data

Methods

n/a	Involved in the study	
☒	☐	ChIP-seq
☐	☒	Flow cytometry
☒	☐	MRI-based neuroimaging

Eukaryotic cell lines

Policy information about cell lines

Cell line source(s)	COLO829 cell line was obtained from American Type Culture Collection (ATCC), Manassas, VA.
Authentication	This melanoma tumor-line has been previously characterized by multiple technologies and provides a benchmark for evaluating somatic alterations, Craig DW et al (2016).
Mycoplasma contamination	Cell line were tested by protocols of the American Type Culture Collection (ATCC), Manassas, VA.
Commonly misidentified lines (See ICLAC register)	We used a well-studied cell-line, COLO829. This cell-line is not present in the ICLAC register for misidentified cell lines.

Flow Cytometry

Plots

Confirm that:

☒ The axis labels state the marker and fluorochrome used (e.g. CD4-FITC).

☒ The axis scales are clearly visible. Include numbers along axes only for bottom left plot of group (a 'group' is an analysis of identical markers).

☒ All plots are contour plots with outliers or pseudocolor plots.

☒ A numerical value for number of cells or percentage (with statistics) is provided.

Methodology

Sample preparation	Cells in the G1 phase were selected for scCNV library preparation.
Instrument	Please see supplemental figures and Methods for detailed description
Software	Please see supplemental figures and Methods for detailed description
Cell population abundance	In Supplementary Figure 1 we describe the Distribution of DNA content in COLO829 nuclei by flow cytometry.
Gating strategy	It was used Vybrant Green*-A. Please see supplemental figure and Methods for detailed description

☒ Tick this box to confirm that a figure exemplifying the gating strategy is provided in the Supplementary Information.
